# Traumatic Brain Injury Patients Admitted on High-Census Days Receive Less Critical Care and Have an Increased Risk for Delirium

**DOI:** 10.7759/cureus.65957

**Published:** 2024-08-01

**Authors:** David R Velez, Anthony J Duncan, Khaled Zreik

**Affiliations:** 1 Department of General Surgery, University of North Dakota School of Medicine and Health Sciences, Fargo, USA; 2 Department of Trauma and Acute Care Surgery, Sanford Medical Center Fargo, Fargo, USA

**Keywords:** delirium, traumatic brain injury, post-traumatic brain injury, patient admission, patient census, impact patient management, icu delirium

## Abstract

Introduction: The utilization of healthcare services in a growing population has raised concerns about its impact on clinical outcomes. Studies have shown that increased hospital census is associated with higher admission rates and unnecessary consults, tests, and procedures in various areas of healthcare. Traumatic brain injuries (TBIs), a significant concern due to their potential for long-term disabilities, are commonly encountered in intensive care units (ICUs) and are a leading cause of patient mortality. Despite extensive research on various aspects of TBI, the effect of the patient census on TBI outcomes remains unexplored. This study aims to investigate the relationship between healthcare provider patient census and clinical outcomes in TBI patients at a level I trauma center.

Methods: A retrospective review was conducted from 2017 to 2022. The mean number of patients per day in the trauma service was determined, with patients below this average considered to be present on low-census days and those above it on high-census days. Patient demographics, mechanisms of injury, vital signs, TBI severity, and associated injuries were analyzed. Adjusted regression analyses were conducted.

Results: Over the study period, 1,527 TBI patients were identified. Demographics were similar between patients admitted on high- and low-census days. Patients with moderate TBI were 30% less likely to be admitted to the ICU on high-census days, whereas there was no difference in ICU admission for patients with mild or severe TBI. Delirium was significantly higher in patients admitted on high-census days compared to those on low-census days. This was further identified to be predominantly driven by patients with mild TBI admitted on high-census days.

Conclusion: While most outcomes remained consistent, significant rates of delirium were found in our mild TBI patients admitted on high-census days suggesting the need for additional factors in the evaluation of these patients on admission. This study also reveals potential under-triage in moderate TBI patients on high-census days as they had significantly lower rates of ICU admission. These findings emphasize the need for further investigations to optimize patient care strategies within the context of fluctuating healthcare system demands.

## Introduction

Researchers have begun investigating the effects of a growing population's increased use of healthcare on clinical outcomes as healthcare utilization continues to surge. An increased patient census in the emergency room has been shown to result in higher admission rates [1]. Furthermore, the increased census of hospital patients has not only led to the ordering of unnecessary consults, tests, and procedures but has also created challenges in effective communication and collaboration among healthcare teams [2]. This increased census can strain physicians, further leading to a decrease in interdisciplinary care [3]. Heavier workloads have also been linked to longer hospital stays and a higher risk of patient safety events [4-6].

In the trauma population, traumatic brain injuries (TBIs) continue to pose a major concern due to their potential to cause long-term disabilities [7-9]. With the primary focus on preventing secondary injury in these patients, quality multidisciplinary rounds are crucial. Patients with TBIs are both a common reason for admission to the intensive care unit (ICU) and a common cause of patient mortality [10-13]. Within this population, a significant number of patients admitted for TBI will develop delirium during their hospitalization with an estimated incidence of 46-69% [14,15]. Studies have suggested an association between the Glasgow Coma Scale and the incidence of delirium [16]. The length of delirium within TBI patients has been shown to be a predictor of decreased long-term functional outcomes [17].

To date, there has been no published data examining the impact of patient census on TBI patients. Our level I trauma center serves a broad catchment area in the upper Midwest, and the patient census can vary significantly throughout the year. Our Trauma and Acute Care Surgery (TRACS) team manages both trauma and emergency general surgery. The team is led by a single fellowship-trained trauma and critical care surgeon with a group of residents and nurse practitioners. This study aims to investigate the interplay between healthcare provider patient census and clinical outcomes.

## Materials and methods

The trauma registry for our level I trauma center was retrospectively reviewed over a five-year time period from July 1, 2017, to June 30, 2022. The daily TRACS census was collected from the electronic health record. The average number of patients on service was determined and deemed to be the cut-off for high- or low-census days. Above-average numbers were defined as a high census with 755 total patients and below-average numbers were defined as a low census with 752 patients. The patient census on the day of admission was then compared to data pulled from our trauma registry.

Background information included demographics, medical comorbidities, and mechanism of injury. We then evaluated the patient’s vital signs on arrival, Glasgow Coma Scale (GCS), Injury Severity Score (ISS), and concomitant injuries. We then examined rates of ICU admission, ICU length of stay, ventilator days, and hospital length of stay. Finally, we examined rates of complications that were listed within our trauma registry and outcomes. For regression analysis, lengths of stay, complications, and outcomes were adjusted for concomitant cervical spine fractures.

Parametric variables were evaluated using the Student's T-test. Qualitative and nonparametric variables were evaluated using the Mann-Whitney U test. The Shapiro-Wilk test was used to confirm a non-normal distribution for hospital length of stay, ICU length of stay, and ventilator days with a significant right skew. Multivariate linear and logistic regression was performed for complications and outcomes, adjusting for concomitant cervical spine fractures. Statistical analyses were performed using Stata version 16.0 (StataCorp LLC, College Station, TX). Tests of significance were two-sided, with p-values considered significant at the 0.05 level.

## Results

Of the 4,696 trauma patients who were seen over this five-year period, 1,527 (32.5%) suffered TBI. The average patient census was 40.9, with a range of 16-70. This mean was used as the cut-off for high- or low-census days. Of those, 775 patients were admitted on days with a high census (greater than 40.9) and 752 were admitted on days with a low census (less than 40.9). There was seasonal variation, and the average patient census was 43.5 from May to October giving more high-census days but 37.2 from November to April (p < 0.001) with more low-census days. Patient demographics and comorbidities were compared in Table 1. TBI patients admitted on high-census days had lower rates of hypertension (31% vs. 39%; p = 0.003). Patients on high-census days also had lower rates of mental health diagnoses (13.8% vs. 18.4%; p = 0.016). There was no other significant difference in patient comorbidities on presentation.

**Table 1:** Comparison of patient demographics and comorbidities for patients with traumatic brain injury admitted on low-census days to those admitted on high-census days. ^1^ Mean (SD), Mann-Whitney U test.^ 2 ^N (%), Student's T-test. * denotes p < 0.05.

	Low census (n = 752)	High census (n = 775)	p-value
Demographics			
Age (years)^1^	55.8 (21.9)	55.0 (21.8)	0.518
Male sex^2^	500 (67.3)	508 (65.5)	0.472
White^2^	626 (83.2)	642 (82.8)	0.833
Black^2^	14 (1.9)	22 (2.8)	0.209
Native American^2^	93 (12.4)	88 (11.4)	0.541
Asian^2^	53 (0.7)	3 (0.4)	0.453
Comorbidities			
Hypertension^2^	293 (39.0)	245 (31.6)	0.003*
Diabetes^2^	116 (15.4)	100 (12.9)	0.158
Hemodialysis^2^	6 (0.8)	7 (0.9)	0.823
Cirrhosis^2^	14 (1.9)	18 (2.3)	0.530
Chronic obstructive pulmonary disease^2^	37 (4.9)	52 (6.7)	0.136
Congestive heart failure^2^	42 (5.6)	51 (6.6)	0.473
Angina^2^	12 (1.6)	24 (3.1)	0.053
Myocardial infarction^2^	6 (0.8)	6 (0.8)	0.959
Peripheral arterial disease^2^	3 (0.3)	5 (0.6)	0.273
Stroke^2^	27 (3.6)	35 (4.5)	0.360
Dementia^2^	36 (4.8)	25 (3.2)	0.120
Mental health diagnosis^2^	138 (18.4)	107 (13.8)	0.016*
Bleeding disorder^2^	18 (2.4)	31 (4.0)	0.075
Anticoagulation^2^	132 (17.6)	128 (16.5)	0.590
Steroid use^2^	11 (1.5)	22 (2.8)	0.065
Chemotherapy^2^	8 (1.1)	13 (1.7)	0.304
Smoker^2^	221 (29.4)	232 (29.9)	0.815
Alcohol abuse^2^	97 (12.9)	81 (10.5)	0.136
Substance abuse^2^	59 (7.8)	60 (7.7)	0.940
Pregnant^2^	1 (0.1)	1 (0.1)	0.984

The comparison of the mechanism of injury between patients admitted on high- and low-census days is shown in Table 2. Patients admitted on high-census days were more likely to have experienced a motorcycle crash and less likely to have been involved in a snowmobile crash.

**Table 2:** Comparison of the mechanism of injury for patients with traumatic brain injury admitted on low-census days to those admitted on high-census days. ^1 ^N (%), Mann-Whitney U test. * denotes p < 0.05.

	Low census (n = 752)	High census (n = 775)	p-value
Fall^1^	386 (51.3)	371 (47.9)	0.177
Motor vehicle crash^1^	161 (21.4)	163 (21.0)	0.857
Motorcycle crash^1^	30 (4.0)	52 (6.7)	0.018*
All-terrain vehicle crash^1^	23 (3.1)	37 (4.8)	0.085
Snowmobile crash^1^	15 (2.0)	2 (0.3)	0.001*
Bicycle crash^1^	11 (1.5)	15 (1.9)	0.476
Pedestrian trauma^1^	19 (2.5)	11 (1.4)	0.119
Sport trauma^1^	2 (0.3)	2 (0.3)	0.977
Assault^1^	46 (6.1)	52 (6.7)	0.637
Gunshot wound^1^	11 (1.5)	9 (1.2)	0.605
Stab wound^1^	0 (0.0)	0 (0.0)	NA

Furthermore, Table 3 reveals that although patients admitted on high-census days had higher rates of cervical spine fracture, there were no other discernible differences in presentation.

**Table 3:** Comparison of presentation and concomitant injuries for patients with traumatic brain injury admitted on low-census days to those admitted on high-census days. ^1^ Mean (SD), Student's T-test. ^2 ^N (%), Mann-Whitney U test. * denotes p < 0.05.

	Low census (n = 752)	High census (n = 775)	p-value
Presentation			
Heart rate (bpm)^1^	85.2 (20.7)	86.5 (21.2)	0.501
Respiratory rate (bpm)^1^	17.7 (4.4)	17.7 (4.2)	0.575
Pulse oximetry^1^	96.3% (3.5)	96.2% (3.7)	0.693
Systolic blood pressure (mmHg)^1^	135.7 (27.2)	136.8 (26.9)	0.410
Temperature (°C)^1^	36.7 (0.7)	36.7 (1.3)	0.179
Injury Severity Score (ISS)^1^	16.5 (10.0)	16.9 (9.9)	0.269
Concomitant injuries			
Rib fracture^2^	166 (22.1)	184 (23.7)	0.404
Skull fracture^2^	175 (23.3)	179 (23.1)	0.936
Facial fracture^2^	155 (20.6)	181 (23.4)	0.196
Cervical spine fracture^2^	99 (13.2)	130 (16.8)	0.048*
Thoracolumbar spine fracture^2^	132 (17.6)	129 (16.6)	0.638
Spinal cord injury^2^	32 (4.3)	30 (3.9)	0.704
Spleen^2^	28 (3.7)	22 (2.8)	0.264
Liver^2^	20 (2.7)	19 (2.5)	0.560
Kidney^2^	21 (2.8)	14 (1.8)	0.237
Pancreas^2^	0 (0.0)	1 (0.1)	0.325
Adrenal^2^	11 (1.5)	9 (1.2)	0.605
Stomach^2^	0 (0.0)	0 (0.0)	NA
Duodenum/small intestine^2^	3 (0.4)	23 (0.3)	0.631
Colon^2^	2 (0.3)	47 (0.6)	0.273
Rectum^2^	0 (0.0)	0 (0.0)	NA
Pulmonary^2^	101 (13.4)	121 (16.3)	0.121
Cardiac^2^	6 (0.8)	8 (1.0)	0.631
Cerebrovascular injury^2^	18 (2.4)	26 (3.4)	0.262
Thoracic vascular injury^2^	5 (0.7)	5 (0.6)	0.962
Abdominal vascular injury^2^	3 (0.4)	1 (0.1)	0.303
Clavicle fracture^2^	40 (5.3)	34 (4.4)	0.397
Scapula fracture^2^	26 (3.5)	40 (5.2)	0.102
Pelvic fracture^2^	42 (5.6)	52 (6.7)	0.361

Similarly, Table 4 illustrates that while high-census days showed lower rates of subdural hematoma, no other notable distinctions were observed. While there were likely some differences within the presentation, these were likely not the result of the hospital census and were more likely driven by seasonal fluctuations.

**Table 4:** Comparison of severity and injury for patients with traumatic brain injury admitted on low-census days to those admitted on high-census days. ^1^ Mean (SD), Mann-Whitney U test. ^2 ^N (%), Mann-Whitney U test. * denotes p < 0.05.

	Low census (n = 752)	High census (n = 775)	p-value
Severity			
Glasgow Coma Scale (GCS)^1^	12.8 (4.1)	12.7 (4.2)	0.732
Mild (GCS 13-15)^2^	595 (79.1)	613 (79.2)	0.960
Moderate (GCS 9-12)^2^	39 (5.2)	27 (3.5)	0.102
Severe (GCS 3-8)^2^	118 (15.7)	134 (17.3)	0.404
Traumatic brain injury			
Concussion^2^	242 (32.2)	228 (29.4)	0.243
Epidural hematoma (EDH)^2^	29 (3.9)	43 (5.5)	0.119
Subdural hematoma (SDH)^2^	361 (48.0)	327 (42.2)	0.022*
Subarachnoid hematoma (SAH)^2^	312 (41.5)	340 (43.9)	0.347
Intraparenchymal hemorrhage (IPH)^2^	168 (22.3)	174 (22.5)	0.959
Cerebellum injury^2^	4 (0.5)	9 (1.2)	0.181
Brainstem injury^2^	6 (0.8)	7 (0.9)	0.823
Diffuse axonal injury (DAI)^2^	25 (3.3)	29 (3.7)	0.659

Adjusting for the cervical spine injury, on low-census days, 53.7% of patients with TBI were admitted to the ICU, while on high-census days, only 48.9% of patients were admitted to the ICU (odds ratio (95% CI) = 0.81 (0.67-0.99), p-value = 0.047). Additionally, these patients had significantly shorter ICU lengths of stay; 3.9 days for patients admitted on high-census days compared to 4.4 days for low-census days (coefficient (95% CI) = -0.64 (-1.27 to -0.02), p-value = 0.044). When stratified by severity, it was seen that patients with moderate TBI were less likely to be admitted to the ICU on high-census days but low and severe TBI patients had no significant difference (Table 5).

**Table 5:** Comparison of ICU admission rates and length of stay for patients with traumatic brain injury admitted on low-census days to those admitted on high-census days, stratified by severity. ^1^ N (%), Student's T-test. ^2^ Mean (SD), Mann-Whitney U test. * denotes p < 0.05. GCS: Glasgow Coma Scale.

	Low census (n = 752)	High census (n = 775)	p-value
ICU admission (%)			
Mild (GCS 13-15)^1^	323 (43.0)	308 (39.8)	0.258
Moderate (GCS 9-12)^1^	598 (79.5)	431 (55.6)	0.040*
Severe (GCS 3-8)^1^	714 (94.9)	700 (90.3)	0.168
ICU length of stay (days)			
Mild (GCS 13-15)^2^	3.3 (5.7)	2.8 (5.1)	0.296
Moderate (GCS 9-12)^2^	5.7 (6.0)	4.6 (3.7)	0.981
Severe (GCS 3-8)^2^	6.4 (3.9)	5.7 (2.9)	0.485

There was no significant difference in ventilator days; 6.4 days for a high census and 5.4 days for a low census (coefficient (95% CI) = -1.28 (-3.07 to 0.50), p-value = 0.158). Similarly, there was no significant difference in hospital length of stay; 6.8 days for a high census and 6.4 days for a low census (coefficient (95% CI) = -0.46 (-1.33 to 0.41), p-value = 0.297).

Complications and outcomes, adjusting for cervical spine injury, were compared in Table 6, each determined by appropriate Current Procedural Terminology (CPT) codes. The majority of outcomes remained the same between patients who were admitted on low- and high-census days. However, patients who presented on high-census days did have higher rates of delirium compared to low-census days (2.7% vs. 1.1%; p < 0.041). Additional breakdown of delirium rates based on TBI severity showed that mild TBI was the driving factor for the statistical difference as patients admitted on low-census days had lower rates of delirium compared to patients admitted on high-census days (0.8%, n = 1 vs. 8.2% n = 11, p = 0.011). In comparison, patients with moderate or severe TBIs had no significant difference in delirium rates (Table 7).

**Table 6:** Comparison of complications and outcomes for patients with traumatic brain injury admitted on low-census days to those admitted on high-census days, adjusted for concomitant cervical spine injury. ^1^ N (%), multivariate logistic regression. * denotes p < 0.05.

	Low census (n = 752)	High census (n = 775)	Adjusted odds ratio (95% CI)	p-value
Complications				
Death^1^	50 (6.6)	57 (7.4)	1.09 (0.73-1.62)	0.668
Any complication^1^	81 (10.8)	73 (9.4)	0.83 (0.59-1.16)	0.266
Delirium^1^	8 (1.1)	21 (2.7)	2.27 (1.04-4.99)	0.041*
Acute kidney injury^1^	7 (0.9)	5 (0.6)	0.62 (0.19-1.98)	0.421
Urinary tract infection^1^	2 (0.3)	3 (0.4)	1.43 (0.24-8.66)	0.691
Acute respiratory distress syndrome (ARDS)^1^	4 (0.5)	1 (0.1)	0.25 (0.03-2.26)	0.218
Pressure ulcer^1^	6 (0.8)	5 (0.6)	0.67 (0.21-2.32)	0.558
Deep venous thrombosis^1^	6 (0.8)	13 (1.7)	2.33 (0.82-6.61)	0.111
Pulmonary embolism^1^	6 (0.8)	5 (0.6)	0.78 (0.24-2.57)	0.681
Stroke^1^	6 (0.8)	3 (0.4)	0.56 (0.13-2.37)	0.434
Myocardial infarction^1^	3 (0.4)	3 (0.4)	0.92 (0.18-4.60)	0.920
Cardiopulmonary resuscitation^1^	10 (1.3)	5 (0.6)	0.60 (0.16-1.37)	0.164

**Table 7:** Comparison of patients suffering delirium based on patient census, stratified by traumatic brain injury severity. ^1^ N/total (%), Student's T-test. * denotes p < 0.05.

	Low census	High census	p-value
Mild^1^	1/118 (0.8%)	11/134 (8.2%)	0.011*
Moderate^1^	1/39 (2.6%)	1/27 (3.7%)	0.809
Severe^1^	7/595 (1.2%)	10/614 (1.6%)	0.505

## Discussion

As the utilization of healthcare continues to rise, the strain on the healthcare system has intensified, particularly in recent years. There also continues to be an increase in geographical population size, density, and increasing mean population survival ages that add to the increased utilization. Increased patient census for healthcare providers has yet to be explored, but our data indicate a potential negative effect on patients presenting with TBI.

Our data showed some seasonal variation. Due to our location in the upper Midwest, our center sees a higher rate of trauma in the summer months. As such, patients, for example, admitted on high-census days in the summer were less likely to have snowmobile crashes but more likely to experience a motorcycle crash. This is consistent with other studies that show the most common mechanism of injury for patients is motorcycle collisions as well as a decrease in these accidents in winter months [18-20]. Patient demographics and presentation were otherwise largely similar between the two groups. The only concomitant injury that had a significant difference was higher rates of cervical spine injury on high-census days, and it was therefore adjusted for during the multivariate analysis.

When adjusting for cervical spine injury, patients admitted on high-census days were significantly less likely to be admitted to the ICU. When stratified by severity, there were no significant differences in ICU admission patterns for patients with mild or severe TBI. Patients with moderate TBI, however, were 30% less likely to be admitted to the ICU. This unexpected finding contradicts the intuitive expectation that physicians tend to escalate care in response to high patient censuses [1]. Within our healthcare system, traumas are initially seen, admitted, and managed by a surgeon with the majority also being board-certified in critical care. Thus, it is not likely attributed to an understanding of ICU requirements, as in the majority of cases, it will be the same faculty both initially seeing the patient and admitting them to the ICU. Our data suggest that patients might be under-triaged on high-census days or over-triaged on low-census days. Patients with mild or severe TBI may have a more concrete admission path, but patients with moderate TBI may be more dependent on physician judgment. Other studies have shown that there is a significant variation between TBI admission levels of care among different hospital settings, which may also be driven by physician variability [21,22]. This seems to be the group at highest risk for under-triage due to potential workload demands.

Overall morbidity and mortality were similar between the two groups. The only significant difference was found in rates of delirium (1.1% (n = 8) for low census compared to 2.7% (n = 21) for high census). Contrary to our initial thinking, when this was further broken down by TBI grade, we found this difference was predominately attributed to patients with mild TBI. We found only 0.8% (n = 1) of patients with mild TBI admitted on low-census days had delirium compared to 8.2% (n = 11) of patients admitted on high-census days (p < 0.011). This was also higher than our patients admitted to the ICU on high-census days in which 1.6% (n = 10) of patients had delirium. While the exact reason for this discrepancy is not clear, this is in contrast to other studies that classically show higher rates of delirium within the ICU [23]. It questions if there are additional factors that are playing a role in these patients developing delirium on the general surgical floor. The higher census may indicate a decrease in direct patient care, as all aspects of the care team are stretched over more patients. Delirium is a critical outcome for TBI patients, as it has been shown to increase long-term cognitive impairment in critically ill patients, raise hospital costs, and elevate six-month mortality [17,24,25].

This study is limited by its retrospective nature and the inherent biases present, including reliance on documentation at the initial presentation. Furthermore, significant changes in global health over the study period, such as the COVID-19 pandemic, are not directly accounted for in this study. However, the large sample size helps to mitigate these limitations. Additionally, this study only examines in-hospital complications and does not address long-term complications that may differ between the populations.

## Conclusions

This study compared the admissions and outcomes of TBI patients admitted on high-census days to those admitted on low-census days. The presentation for most patients was similar, with some seasonal variation in mechanisms. However, patients admitted on high-census days were less likely to be admitted to the ICU. Patients with moderate TBI were particularly at risk for potential under-triage, as evidenced by decreased rates of ICU admission. Delirium rates were significantly higher in mild TBI patients admitted on high-census days, raising the question of whether additional variables should be evaluated upon admission to help decrease delirium rates. All other measures of morbidity and mortality were similar between the two groups. These data underscore the need to maintain the quality of care as workloads increase. Physicians and hospitals must anticipate these changes and be able to adapt to high-volume periods.
